# Supplementary material for: A diffusion of innovations measurement scale for reinvention, relative advantage, compatibility, complexity, trialability and observability
Source: PLoS One. 2025 Oct 16;20(10):e0334616. doi: 10.1371/journal.pone.0334616 (PMC12530589; doi:10.1371/journal.pone.0334616)
Supplement: S1 Table — The final validated scale items for measuring all six DOI attributes. (DOCX) [file pone.0334616.s001.docx]

**Final Diffusion of Innovations Scale**

**S1 Table 1. Final Diffusion of Innovations Scale.**

| **Relative Advantage** | **Compatibility** | **Complexity** | **Trialability** | **Observability** | **Reinvention** |
| --- | --- | --- | --- | --- | --- |
| [Innovation] allows me to accomplish tasks such as [task] more efficiently | [Innovation] fits well with the way that I like to [task] | It is easy to get [innovation] to do what I want them to [task] | I have the ability to try out [innovation] to accomplish [task] before deciding whether I like it or not | I am able to observe when others in my environment use [innovation] to [task] | I often have to experiment with new ways of using [innovation] |
| [Innovation] is the best way to accomplish [task] | [Innovation] is completely compatible with my current way of [task] | Learning to operate [innovation] to accomplish [task] is easy for me | Trying out [innovation] to accomplish [task] has informed my decision to use [innovation] | My friends are able to observe the results of using [innovation] | I often have to modify [innovation] to get it to work for me |
| Using [innovation] helps me accomplish [task] better than not using [innovation] | [Innovation] suits my needs when [task] | My interactions with [innovation] is clear and understandable | I have had the opportunity to try [innovation] in the past | Others in my environment notice the impact of using [innovation] to [task] | I adapt [innovation] in a way that is different from how it was originally intended to be used |
|  | [Innovation] integrates well with my current way of using [technology] |  |  |  |  |
