# Supplementary material for: A diffusion of innovations measurement scale for reinvention, relative advantage, compatibility, complexity, trialability and observability
Source: PLoS One. 2025 Oct 16;20(10):e0334616. doi: 10.1371/journal.pone.0334616 (PMC12530589; doi:10.1371/journal.pone.0334616)
Supplement: S2 File — The list of initial items used to measure all six DOI attributes across all contexts. (DOCX) [file pone.0334616.s002.docx]

**Initial Items used to make adaptable diffusion of innovations scale**

**Facial Recognition**

Think about facial recognition software, such as FaceID or other facial recognition phone unlock features, **which allows users to unlock their phone by scanning and verifying their face**. Facial recognition phone unlock means that instead of a fingerprint or a PIN, the phone uses its camera to recognize a user’s face and unlock the device.

**S2 Table 1. Facial recognition.**

| **Relative Advantage** | **Compatibility** | **Complexity** | **Trialability** | **Observability** | **Reinvention** |
| --- | --- | --- | --- | --- | --- |
| Facial recognition technology allows (would allow) me to accomplish tasks, such as unlocking my phone, more efficiently | Facial recognition technology fits (would fit) well with the way that I like to use my phone | It is (would be) easy to get facial recognition algorithms to do what I want them to do when using them to unlock a phone | I have (anticipate having) the ability to try out facial recognition technology to unlock a phone before deciding whether I like it or not. | Changes in others’ use of facial recognition technology to unlock their phone (would be) obvious to me | I often have (anticipate having) to experiment with new ways of using facial recognition technology when using it to unlock my phone |
| Facial recognition is (would be) the best way to unlock my phone | Facial recognition technology is (would be) completely compatible with my current way of using my phone | Learning to operate facial recognition technology to unlock a phone is (would be) easy for me | Trying facial recognition to unlock a phone has informed my decision to use facial recognition to unlock a phone | I am (would be) able to observe when others in my environment use facial recognition technology to unlock a phone | I often have (anticipate having) to modify facial recognition technology to get it to work for me when unlocking my phone |
| The disadvantages of using facial recognition technology to unlock my phone (would) outweigh the advantages | Facial recognition technology suits (would suit) my needs when unlocking my phone | My interaction with facial recognition technology used to unlock a phone is (would be) clear and understandable | I have or have had the opportunity to try facial recognition to unlock a phone in the past | My friends are (would be) able to observe the results of using facial recognition technology to unlock a phone | I adapt (anticipate having to adapt) facial recognition technology in a way that is different from how it was originally intended to be used when unlocking my phone |
| Using facial recognition technology helps (would help) me unlock my phone better than not using facial recognition | [Facial recognition technology integrates (would integrate) well with my current way of using my phone | Using facial recognition to unlock a phone is (would be) cumbersome | I have not had much opportunity to try facial recognition to unlock a phone in the past | Others in my environment (would) notice the impact of using facial recognition technology to unlock a phone | I rarely have (anticipate having) to come up with novel ways to get facial recognition technology to work for me when using it to unlock my phone |

**Finances**

Now, think about facial recognition software that allows users to **access their account on a mobile financial application**, such as an application for a bank account. Facial recognition in this case means that instead of a password or a fingerprint, the phone uses its camera to recognize a user’s face and unlock the account.

**S2 Table 2. Finances.**

| **Relative Advantage** | **Compatibility** | **Complexity** | **Trialability** | **Observability** | **Reinvention** |
| --- | --- | --- | --- | --- | --- |
| Facial recognition technology allows (would allow) me to accomplish tasks, such as checking my bank account, more efficiently. | Facial recognition technology fits (would fit) well with the way that I like to use technology to check my financial accounts | It is (would be) easy to get facial recognition algorithms to do what I want them to do when checking my financial accounts | I have (anticipate having) the ability to try out facial recognition technology to check my financial accounts before deciding whether I like it or not | Changes in others’ use of facial recognition technology to check their financial accounts (would be) obvious to me | I often have (anticipate having) to experiment with new ways of using facial recognition technology |
| Facial recognition is (would be) the best way to access a bank account. | Facial recognition technology is (would be) completely compatible with my current way of using technology to check my financial accounts | Learning to operate facial recognition technology to access my financial accounts is (would be) easy for me | Trying facial recognition technology to check my financial accounts has informed my decision to use facial recognition algorithms | I am (would be) able to observe when others in my environment use facial recognition technology to check their financial accounts | I often have (anticipate having) to modify facial recognition technology to get it to work for me |
| The disadvantages of using facial recognition technology to access a bank account (would) outweigh the advantages | Facial recognition technology suits (would suit) my needs when accessing my financial accounts | My interaction with facial recognition technology to access my finances is (would be) clear and understandable | I have or have had the opportunity to try facial recognition to check my financial accounts in the past | My friends are (would be) able to observe the results of using facial recognition technology to check financial accounts | I adapt (anticipate having to adapt) facial recognition technology in a way that is different from how it was originally intended to be used |
| Using facial recognition technology helps (would help) me accomplish tasks, such as checking my bank account, better than not using facial recognition | Facial recognition technology integrates (would integrate) well with my current way of using technology to check my financial accounts | Using facial recognition to access my financial accounts is (would be) cumbersome | I have not had much opportunity to try facial recognition to check my financial accounts in the past | Others in my environment (would) notice the impact of using facial recognition technology to check financial accounts | I rarely have (anticipate having) to come up with novel ways to get facial recognition technology to work for me |

**Social Media**

Now, think about **facial filters** such as those used on applications like TikTok and Instagram. **Facial filters are used to create effects on the face**, such as beautifying or adding a silly mask.

**S2 Table 3. Social media.**

| **Relative Advantage** | **Compatibility** | **Complexity** | **Trialability** | **Observability** | **Reinvention** |
| --- | --- | --- | --- | --- | --- |
| Facial recognition technology allows (would allow) me to accomplish tasks on social media more efficiently | Facial recognition technology fits (would fit) well with the way that I like to use social media | It is (would be) easy to get facial recognition algorithms on social media to do what I want them to do | I have (anticipate having) the ability to try out facial recognition technology on social media before deciding whether I like it or not | Changes in others’ use of facial recognition technology such as social media facial filters (would be) obvious to me. | I often have (anticipate having) to experiment with new ways of using facial recognition technology such as social media facial filters |
| Facial recognition is (would be) the best way to use social media | Facial recognition technology is (would be) completely compatible with my current way of using social media | Learning to operate facial recognition technology on social media is (would be) easy for me | Trying facial recognition algorithms on social media has informed my decision to use facial recognition algorithms. | I am (would be) able to observe when others in my environment use facial recognition technology such as social media facial filters | I often have (anticipate having) to modify facial recognition technology such as social media facial filters to get it to work for me |
| The disadvantages of using facial recognition technology on social media (would) outweigh the advantages | Facial recognition technology suits (would suit) my needs when using social media | My interaction with facial recognition technology on social media is (would be) clear and understandable | I have or have had the opportunity to try facial recognition algorithms on social media in the past. | My friends are (would be) able to observe the results of using facial recognition technology such as social media facial filters | I adapt (anticipate having to adapt) facial recognition technology such as social media facial filters in a way that is different from how it was originally intended to be used |
| Using facial recognition technology helps (would help) me accomplish tasks on social media better than not using facial recognition | Facial recognition technology integrates (would integrate) well with my current way of using social media | Using facial recognition on social media is (would be) cumbersome | I have not had much opportunity to try facial recognition algorithms on social media in the past | Others in my environment (would) notice the impact of using facial recognition technology such as social media facial filters | I rarely have (anticipate having) to come up with novel ways to get facial recognition technology such as social media facial filters to work for me |

**Image Sensors**

Now, think about **image sensors** that you encounter in daily life. These sensors are used in a wide variety of daily applications, including automatic water faucets that dispense water when you place your hands under the faucet, automatic paper towel dispensers that dispense paper towels when you wave your hand in front of the sensor, and automatic soap dispensers that dispense soap when you place your hand by the sensor.

**S2 Table 4. Image sensors.**

| **Relative Advantage** | **Compatibility** | **Complexity** | **Trialability** | **Observability** | **Reinvention** |
| --- | --- | --- | --- | --- | --- |
| Image sensors allow (would allow) me to complete my tasks more quickly | Using image sensors (would fit) well with the way that I like to use technology | It is (would be) easy to get image sensors to do what I want them to do. | I have (anticipate having) the ability to try out image sensors before deciding whether I like them or not | Changes in others' use of image sensors (would be) obvious to me | I often have (anticipate having) to experiment with new ways of using image sensing technology |
| Image sensors are (would be) the best way to complete tasks | Using image sensors is (would be) completely compatible with my current way of using technology | Learning to operate image sensing technology is (would be) easy for me | Trying image sensors has informed my decision to use image sensors | I am (would be) able to observe when others in my environment use image sensors | I often have (anticipate having to) to modify image sensing technology to get it to work for me |
| The disadvantages of using image sensors (would) outweigh the advantages | Using image sensors suits (would suit) my needs | My interaction with image sensing technology is (would be) clear and understandable | I have or have had the opportunity to try image sensing algorithms in the past | Other friends (would be) able to observe the results of using image sensors | I have adapted (anticipate having to adapt) image sensing technology in a way that is different from how it was originally intended to be used |
| Using image sensors (would help) me accomplish tasks better than not using image sensors | Using image sensors integrates (would integrate) well with my current way of using technology | Using image sensing technology is (would be) cumbersome | I have not had much opportunity to try image sensing algorithms in the past | Others in my environment (would) notice the impact of using image sensors | I rarely have (anticipating having) to come up with novel ways to get image sensing technology to work for me |
